# *MALINTO*: A New MALDI Interpretation Tool for Enhanced Peak Assignment and Semiquantitative Studies of Complex Synthetic Polymers

**DOI:** 10.1021/jasms.2c00311

**Published:** 2023-01-04

**Authors:** Klara M. Saller, Daniel C. Pernusch, Clemens Schwarzinger

**Affiliations:** † Institute for Chemical Technology of Organic Materials, Johannes Kepler University Linz, Altenbergerstrasse 69, 4040Linz, Austria

## Abstract

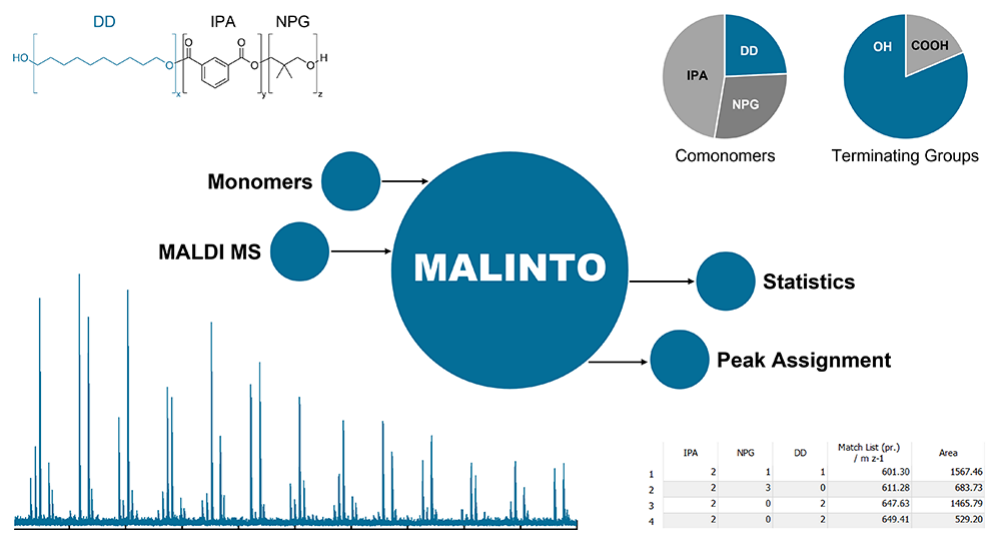

The newly developed MALDI interpretation tool (“*MALINTO*”) allows for the accelerated characterization of complex synthetic polymers via MALDI mass spectrometry. While existing software provides solutions for simple polymers like poly(ethylene glycol), polystyrene, etc., they are limited in their application on polycondensates synthesized from two different kinds of monomers (e.g., diacid and diol in polyesters). In addition to such A_2_ + B_2_ polycondensates, *MALINTO* covers branched and even multicyclic polymer systems. Since the *MALINTO* software works based on input data of monomers/repeating units, end groups, and adducts, it can be applied on polymers whose components are previously known or elucidated. Using these input data, a list with theoretically possible polymer compositions and resulting *m*/*z* values is calculated, which is further compared to experimental mass spectrometry data. For optional semiquantitative studies, peak areas are allocated according to their assigned polymer composition to evaluate both comonomer and terminating group ratios. Several tools are implemented to avoid mistakes, for example, during peak assignment. In the present publication, the functions of *MALINTO* are described in detail and its broad applicability on different linear polymers as well as branched and multicyclic polycondensates is demonstrated. Fellow researchers will benefit from the accelerated peak assignment using the freely available *MALINTO* software and might be encouraged to explore the potential of MALDI mass spectrometry for (semi)quantitative applications.

Originally developed for the analysis of biomolecules,^1,2^ matrix assisted laser desorption/ionization (MALDI) mass spectrometry (MS) soon attracted the interest of polymer analysts since the technique reveals information on molecular mass distributions, terminating groups, and the composition of blends or copolymers.^3,4^ The introduction of MALDI especially influenced the field of polycondensation chemistry because it allowed the direct proof of ring formation for the first time.^5^ This discovery still affects current research and leads to the continuous reformation of classic polycondensation theories, mostly driven by Kricheldorf et al.^6−8^ Further recent studies show the increasing importance of MALDI for the characterization of complex polymer structures^9−12^ as well as the ongoing development of MALDI techniques.^12−14^

While MALDI is highly appreciated as a tool for identification of various macromolecules and mixtures thereof, quantification faces several disadvantages. These include suppression of higher molecular weights, limitation of resolution for broader *m*/*z* regions, varying ionization efficiencies of different species, and dependence on sample preparation.^15−18^ However, several studies on particular polymeric systems already show successful semiquantitative approaches,^17,19,20^ a development which could make MALDI MS an even more powerful tool for advanced polymer analysis.

Parallel to the ongoing research on MALDI applications, processing of data is a crucial topic. Although a lot of information can be extracted from MALDI mass spectra, manual evaluation is time-consuming and tedious. Therefore, various suppliers of MALDI mass spectrometers (Bruker, Jeol, Shimadzu, Waters, etc.), as well as specialized software companies (e.g., Sierra Analytics), offer programs for MALDI MS interpretation. Additionally, individual research groups have published free software tools with focus on copolymer composition,^21,22^ end group determination,^23^ imaging,^24−26^ MS/MS,^23,27−29^ and hyphenations.^30,31^ The Kendrick analysis, thoroughly reviewed by T. Fouquet,^32^ became a common tool for the qualitative interpretation of mass spectra.

Nevertheless, neither program description seems to face the challenges provided by complex polyaddition or -condensation products. To evaluate such systems in a faster, semiautomated way, we have developed a new MALDI interpretation tool “*MALINTO*”, which was tested on linear (co)polymers, as well as on branched and multicyclic polyesters. Using input data for monomers, end groups, and adducts, *MALINTO* calculates theoretical mass lists which provide possible polymer compositions for experimental peak data. After peak assignment, peak areas can be used for quantitative analyses of the MALDI spectra. *MALINTO* significantly accelerates interpretation of mass spectra, therefore enabling a higher number of experiments, which is needed to test more potential (semi)quantitative applications of MALDI including sufficient data on reproducibility.

## Experimental Section

### Software Development

Based on the needs for peak assignment and quantitative evaluation of complex polymer systems using MALDI mass spectrometry, the *MALINTO* program was designed and written in GNU Octave, which is an open source software. Version 6.4.0 has been used for the development of the MALDI interpretation tool. In a first step, *MALINTO* is provided with the expected masses and functionalities of up to 4 monomers (repeating units), 10 end groups, and 4 cations (adducts). Second, a theoretical mass list is generated including all possible combinations of the input data up to a certain molecular mass or number of repeating units. Third, this theoretical mass list is compared to the Excel mass list of a MALDI experiment which includes *m*/*z* ratios and corresponding peak areas. In this case, Excel mass lists are generated by the Bruker FlexAnalysis software, but similar exports from varying instruments can be used as long as formatting requirements are met. After checking the assignments, peak data are used for the calculation of comonomer and terminating group ratios. More details are found in the Results and Discussion section as well as the step-by-step program manual provided in the *MALINTO* software package, which can be downloaded using the following link: https://www.jku.at/en/institute-for-chemical-technology-of-organic-materials/publications/software/malinto/ (accessed on December 15, 2022).

### MALDI-ToF MS

Unless otherwise stated, MALDI mass spectra were recorded using the Bruker autoflex III smartbeam in reflectron mode. Solutions of matrix (10 mg mL^–1^), sample (10 mg mL^–1^), and ionization agent (1 mg mL^–1^) were prepared, mixed in a 100:10:1 ratio, and applied to the MALDI target via dried droplet method. Combination of solvent, matrix, and salt depended on the individual analytes and are summarized in Table S2 in the Supporting Information. 2,5-Dihydroxybenzoic acid (DHB, Sigma, >99%), *trans*-2-[3-(4-*tert*-butylphenyl)-2-methyl-2-propenylidene]malononitrile (DCTB, abcr, 98%), and dithranol (MP Biomedicals, recrystallized in ethanol) were used as matrices, and sodium trifluoroacetate (Fluka, 99%), potassium trifluoroacetate (Aldrich, 98%), and silver perchlorate (Aldrich, 97%) were used as ionization agents. Spectra were processed using the Bruker FlexAnalysis 3.0 software which generated mass lists of deconvoluted peaks including peak areas. MALDI data on multicyclic polyesters of trimesic acid and alkanediols were kindly provided by Steffen Weidner and previously published.^33^

### Polymer Synthesis, Materials, and Further Characterization

The examples given at the end of the Results and Discussion section and in the Supporting Information demonstrate the range of applications of the *MALINTO* program. This includes spectrum interpretation of linear polyesters (PES) like poly(lactic acid) (PLA), branched polyesters (bPES), and multicyclic polyesters (cPES), as well as polystyrene (PS) as example for a chain-growth polymer. Due to the high number of different samples, materials, preparation, and characterization methods of the individual polymer systems are summarized in the Supporting Information and partly given in the literature.^17,34^

## Results and Discussion

### Software Development

The *MALINTO* software aims to facilitate and accelerate the (semiquantitative) interpretation of MALDI spectra of complex samples. Input data include the mass and kind of monomers, expected end groups, and adducts. The software calculates theoretical *m*/*z* values by using all mathematically possible combinations of these input data. By defining different kinds of functionalities present in the monomers (e.g., COOH, OH, NCO), chemically preposterous structures such as reaction products of two isocyanates are excluded. For the semiquantitative analysis of experimental data, *m*/*z* values and corresponding areas of the deconvoluted peaks are required, which are for example exported from the Bruker FlexAnalysis software. Comparing the experimental *m*/*z* values to the theoretical mass list gives possible assignments of the measured peaks. Ambiguous or potentially wrong assignments are highlighted (multiple match column, deviation graph) and have to be corrected manually. The statistic tool filters the cleaned up data according to comonomer composition and terminating groups. Separate results for varying chain lengths are shown both numerically and graphically to observe inaccuracies or trends. After the possibility to delete outliers, mean value and standard deviation for comonomer composition and terminating groups are given as final results. A step-by-step manual including respective illustrations of the software can be found in the *MALINTO* download package.

**Scheme 1:**
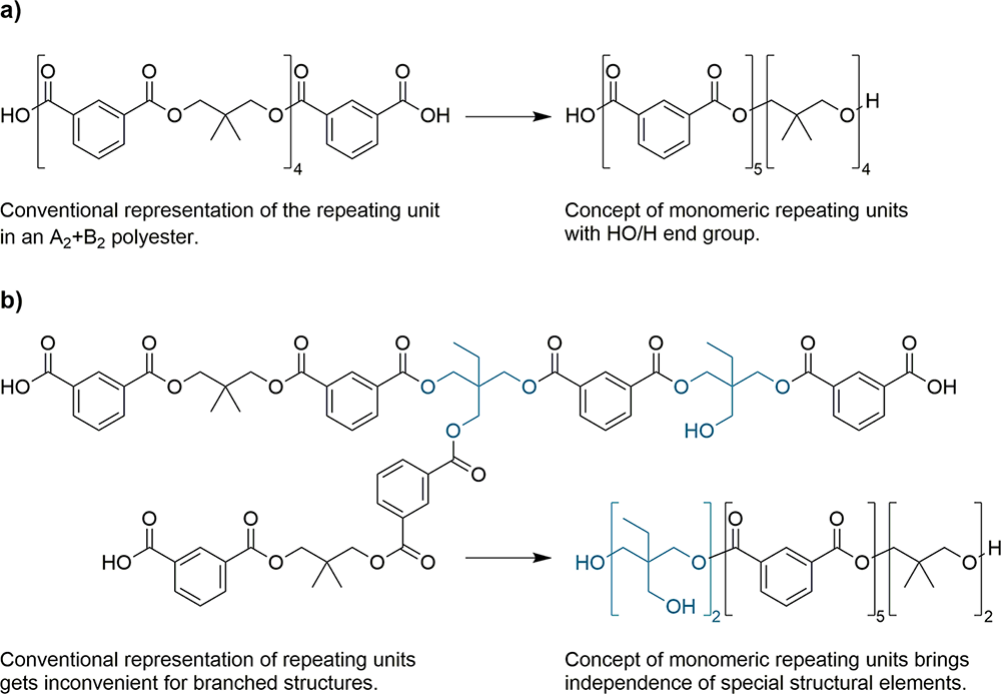
Concept of Monomeric Repeating Units Is Independent from Structural Features and Can Be Applied on Both (a) A_2_ + B_2_ Polycondensates (e.g., Isophthalic Acid/Neopentyl Glycol) and (b) Branched Polycondensates (e.g., with Trimethylolpropane) while the Conventional Representation Becomes Inconvenient for the Latter

The software was originally designed for the analysis of A_2_ + B_2_ polyesters, which are synthesized using at least one diacid (A_2_) and one diol (B_2_). In this case, a repeating unit consists of two different monomers as shown in Scheme 1a. However, the representation of such combined repeating units soon gets complicated if, for example, branched monomers are included (Scheme 1b). Hence, it was preferred to use monomeric repeating units which can generally be applied to all kind of different structures. Scheme 1 includes such conceptional polymer structures, and it can be seen that a monomeric repeating unit consists of the monomer which optionally lost a condensation product (e.g., water). In the shown example, the masses of isophthalic acid (166.03 Da), trimethylolpropane (134.09 Da), and neopentyl glycol (104.08 Da) are thus reduced by 18.01 Da for a water molecule to give the monomeric repeating units with masses of 148.02, 116.08, and 86.07 Da, respectively. Each of these monomeric repeating units is linked to one or two functionalities (COOH, OH) which by definition can only react with their counterparts and not with themselves.

Additional to the repeating units of a polymer, different end groups can be observed depending on the individual reaction mechanisms and structures. Ring-opening and anionic polymerizations are for example started by using different kinds of initiators which are chemically linked to the polymer backbone, thus affecting the polymers’ molecular weight. The same accounts for functionalization with different terminating agents, as for example in anionic polymerizations. Step-growth polymerization reactions on the other hand do not require activation by initiators. Different terminating groups occur due to different degrees of condensation and different kinds of monomers. If an A_2_ + B_2_ polyester is fully condensed, a cyclic structure is obtained which only consists of the monomeric repeating units. For the identification of these species, the end group is defined with a mass of 0 Da. Further examples of ring structures are explained in example 3, which describes the structures of multicycles synthesized using a branching monomer. If polyesters or similar polycondensates have linear structures, the formal end groups equal the products cleaved off during condensation (HO/H, Cl/H, CH_3_O/H, ...) which are not part of the monomeric repeating units (Scheme 1).

Such formal end groups like HO/H do not thoroughly describe the structure of polycondensates since the actual terminating groups are carboxylic acids, acyl chlorides, alcohols, isocyanates, amines, etc. These “free functionalities” which significantly influence polymer properties and allow further reactions are, however, already defined by the number of the individual monomeric repeating units and corresponding functionalities in a polymer species. Thus, information on free functionalities is extracted directly from the peak assignment and displayed in the separate “Free Functionality Statistics”.

**Figure 1:**
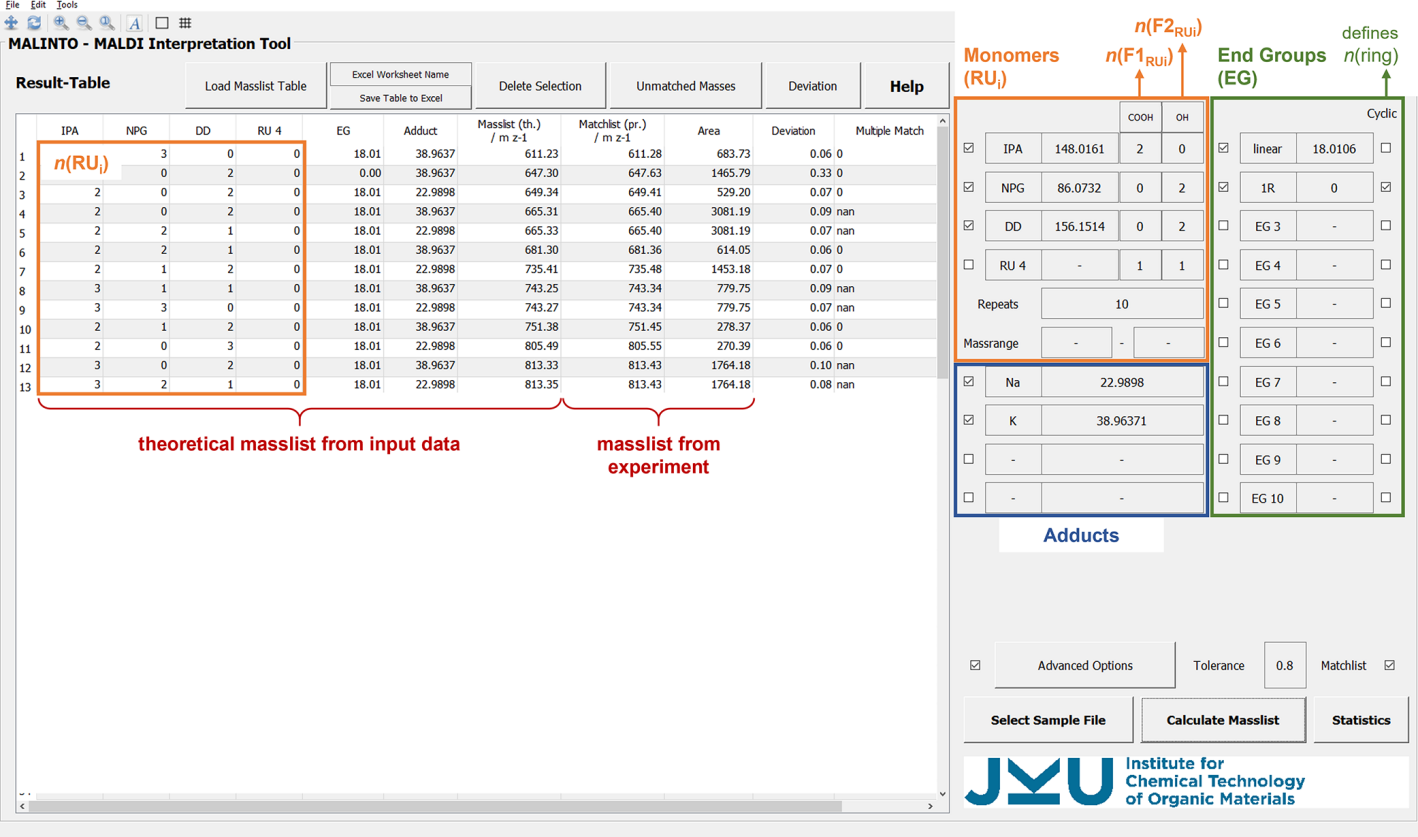
Overview of the *MALINTO* program. The input data including monomeric repeating units, end groups, and adducts are found on the right side. These entries are used by *MALINTO* to generate a theoretical mass list which can further be compared with experimental data. In order to exclusively obtain realistic chemical compositions, 3 conditions are defined using details on the kind and number of functionalities of a repeating unit (*n*(F1_RUi_), *n*(F2_RUi_)), number of rings in a polymer species (*n*(ring)), etc.

An overview of the *MALINTO* software is given in Figure 1. After definition of the input data (monomeric repeating units (RU_i_) including number and kind of functionalities, end groups (EG), and adducts), *MALINTO* calculates the theoretical mass list by combining those input data according to eq 1, where *m* represents the masses of the individual components.
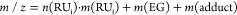
1

While the monomeric repeating unit approach is independent from structural features and thus expands the field of applications significantly, several results in the theoretical mass list would not represent realistic chemical structures (e.g., reaction product of two carboxylic acids). Thus, each mathematically possible polymer composition is checked to fulfill 3 conditions in order to appear in the resulting mass list table. The application of these conditions is demonstrated in the Excel file “Example_MassLists_Comments”, which is part of the *MALINTO* download package.

1. A functional group F1 only reacts with functional groups of different kinds (F2) and vice versa. Hence, both F1 and F2 have to be present in the polymer composition which is expressed in eq 2.
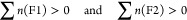
2∑ *n*(F1) and ∑ *n*(F2) are calculated using eqs 3–4 in which *n*(F1_RUi_) and *n*(F2_RUi_) represent the number of functionalities of a first and second kind in a repeating unit RU_i_ (Figure 1). *n*(RU_i_) is the number of respective repeating unit in the polymer composition.
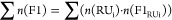
3
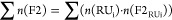
4Combinations of functional groups are, for example, COOH/OH for polyesters, COOH/NH_2_ for polyamides, and NCO/OH for polyurethanes. In case of polystyrene, polyacrylates, etc., both F1 and F2 columns are filled with the amount of present double bonds (e.g., *n*(F1_styrene_) = *n*(F2_styrene_) = 1, *n*(F1_divinylbenzene_) = *n*(F2_divinylbenzene_) = 2).
2. The excess of one functionality is limited since the other functionality is needed for further reaction. This limit is the number of free functionalities *n*(FF) present in the polycondensate as shown in eqs 5–6.
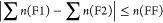
5
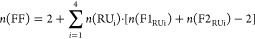
6
  The number of free functionalities is calculated as the deviation from the linear case in which the term in the square bracket is 0 and *n*(FF) is 2. Branching occurs if one or more monomers have more than 2 functional groups, independent of its kind (F1 or F2). Applying eq 6 on the example with trimethylolpropane (TMP) in Scheme 1 gives a result of 4 free functionalities, which is confirmed by the shown structure (3 COOH, 1 OH).
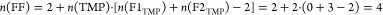
3. Cyclic structures can only form when both functional groups are present as free functionalities, which is calculated by the total number of free functionalities and functionality ratio (eqs 7–9). The software only tests for this condition if “cyclic” is selected next to the defined end groups in the input data (Figure 1, top right).
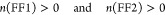
7
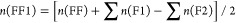
8
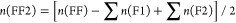
9

As shown by Kricheldorf and Weidner, multiple cyclization events may occur in branched polycondensates.^33^ In this case “end groups” with negative masses due to continuing condensation reactions are defined to calculate the correct *m*/*z* values (−18.01 Da for 2 rings, −36.02 Da for 3 rings, etc.). Again, the number of rings which can be formed depends on the number of free functionalities as shown in eq 10.
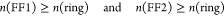
10

For the correct evaluation of free functionalities in the statistics tool, the numbers of free functionalities (eq 11), FF1, and FF2 have to be corrected since 2 functional groups are consumed by the formation of a ring.
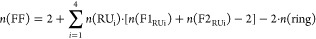
11

**Figure 2:**
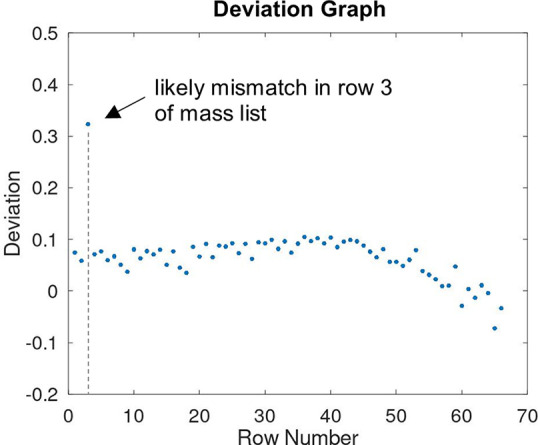
After assigning experimental MALDI peaks, the deviation of the measured to the theoretical *m*/*z* value indicates potential mismatches.

The resulting theoretical mass list is sorted by *m*/*z* values and can already be used for quickly identifying peaks in the MALDI mass spectrum during measurements. After gaining experimental data, Excel mass lists which are in this case generated by the Bruker FlexAnalysis software are uploaded to the software. Within an adjustable tolerance range peaks are assigned to theoretical *m*/*z* values and corresponding polymer compositions. Depending on the accuracy and range of calibration, the deviation of the experimental to the theoretical values should be relatively constant. Graphically presented in the “Deviation Graph” (Figure 2), this can be used for the identification of potentially wrong assignments.

Investigating complex polymer structures with a high number of different repeating units, end groups, and adducts leads to an exponential increase of theoretical *m*/*z* values within a certain mass range. Several polymer species and adduct combinations give similar molecular weights, and assignment of experimental peaks is ambiguous. The “Multiple Match” column highlights such peaks that then have to be manually assigned. The polymer system should be well-known in this case to avoid severe mistakes. In example 1, results for exclusively interpreting the dominant adduct species (Na) are in good agreement with results including all 4 adducts. Investigating for example a four-component polyester with IPA, NPG, 1,10-decanediol, and fumaric acid gives 15 different polymer compositions in the *m*/*z* range of 2000–2010 if only sodium adducts are considered. Including further adducts, the number of possible combinations within a certain mass range multiplies by the number of adducts, which makes spectrum interpretation very difficult to impossible.

**Figure 3:**
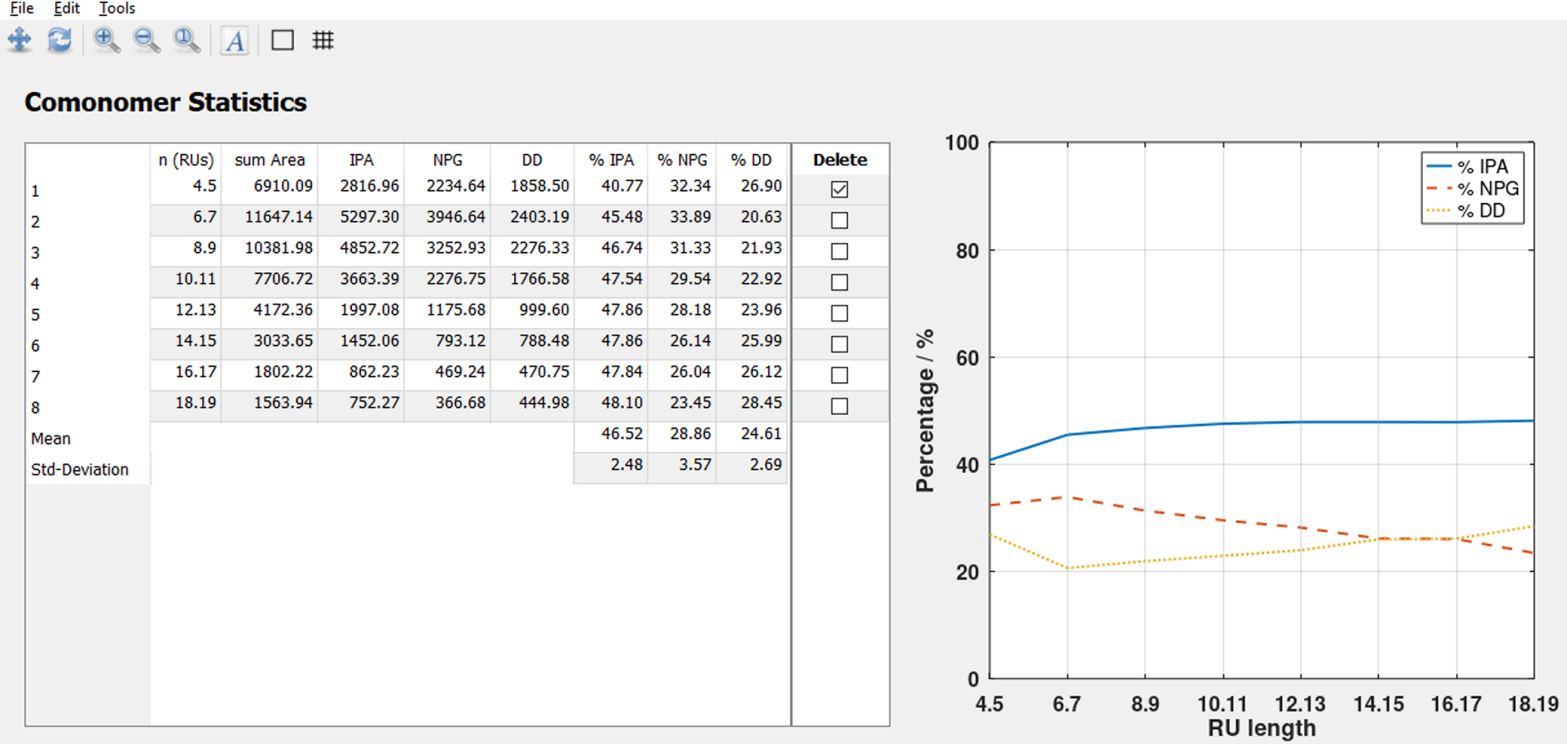
Comonomer statistics of a copolyester sample containing isophthalic acid (IPA), neopentyl glycol (NPG), and 1,10-decanediol (DD). Individual peaks are summarized according to their chain length, and the peak area is split between present comonomers. The graph on the right helps to interpret the reliability of the results and shows a slightly increasing DD content at higher chain lengths.

The statistics tool is applied after removal of potentially wrong or ambiguous assignments. If more than 1 adduct has been used for the interpretation, peak areas for the same polymer species are summed up, after which areas are split according to their assigned polymer composition to evaluate both comonomer and terminating group ratios. In order to introduce a control unit which indicates inaccuracies or trends, results are shown for different chain lengths (Figure 3). Two consecutive numbers of repeating units *n*(RU) are summarized, because in the case of polycondensates all even numbers will give mixed terminated or cyclic species, while the excess of one component leads to an odd *n*(RU). Depending on the kind of statistics, results are filtered according to comonomers (kind of repeating units), end groups, or free functionalities which are derived from the polymer composition using eqs 8 and 9. The correction of the number of free functionalities in the case of ring formation is considered. After the possibility to delete outliers, the final results will show in a separate window. After each step, input data, mass or match lists, and statistical evaluations can be exported as an Excel file.

*MALINTO* can be applied on very different polymer systems, some of which are presented in the following examples. At this point it shall again be noted that quantification by MALDI mass spectrometry is critical due to several reasons, such as discrimination of higher masses, different ionizabilities of monomers and terminating groups, inhomogeneous sample spots, etc. Nevertheless, previous results have shown that there are systems for which MALDI can be used as a semiquantitative tool.^17,35^ The *MALINTO* software significantly accelerates such studies and shall motivate researchers to test this potential of MALDI mass spectrometry further.

### Example 1: A_2_ + B_2_ Homo- and Copolyesters

**Figure 4:**
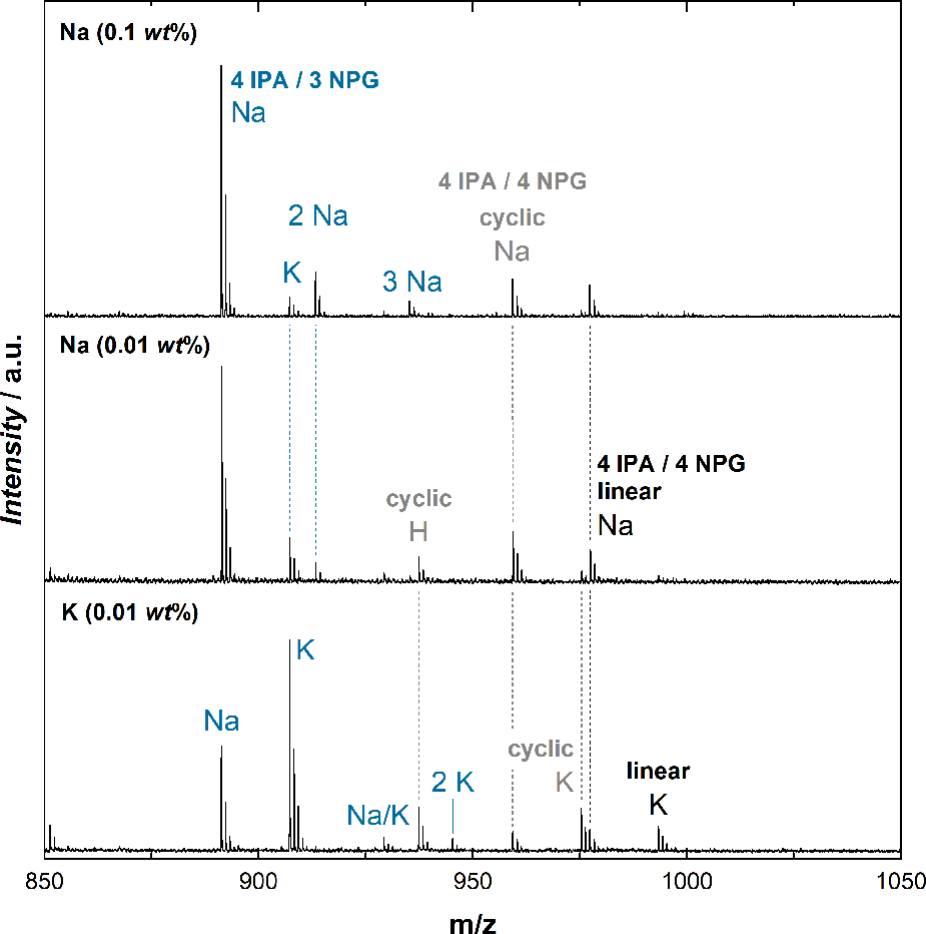
Multiple adducts and deprotonation of carboxylic groups are observed for IPA-NPG homopolyesters (PES1), depending on the kind and concentration of the ionizing agent.

The detail of a MALDI mass spectrum (*m*/*z* = 850–1050) of an IPA-NPG homopolyester with an excess of isophthalic acid (PES1) is shown in Figure 4. Using sodium trifluoroacetate as ionization agent (0.01–0.02 wt % of polyester) led to the observation of different adducts. Cyclic species were ionized by H, Na, and K adducts, whereas only Na and K adducts were observed for linear species. If carboxyl acid free functionalities were present, the formation of sodium carboxylates led to an additional combination (“2Na”) which could be treated like the other adducts. With the idea of simplifying spectrum interpretation by reducing the occurrence of different adduct species, the kind and amount of ionization agents were varied. Significantly increasing the amount of sodium salt to 0.1 wt % suppressed the cyclic H peak but led to further “3Na” adducts for polyesters with 2 acid terminating groups, and potassium adducts were still observed. Switching to potassium trifluoroacetate did not reduce the number of different adducts either, and the K/Na ratio of peak areas was significantly smaller than the Na/K ratio when using sodium trifluoroacetate.

Although modifications of salt and salt concentration led to similar results for the fraction of free acidic functionalities (%FF COOH = 93–96% with standard deviations of 2–3%), these simple variations did not reduce the number of different adducts. Thus, the concentration of sodium trifluoroacetate was kept at 0.01 wt %, and it was tested to consider only the dominant sodium peaks for the interpretation of the MALDI mass spectra. Although the number of assigned peaks drastically decreased from 64 to 30 for PES1, results for %FF COOH were equal as seen in Table 1.

**Table 1:** Investigation of the Acidic Free Functionalities (%FF COOH) in Homo- (PES1) and Copolyesters (PES2–5)^a^

	%FF COOH	
sample	4 adducts	Na
PES1	94 ± 3	94 ± 1
PES2	97 ± 1	97 ± 1
PES3	95 ± 2	95 ± 1
PES4	99 ± 3	99 ± 3
PES5	92 ± 2	93 ± 1

a Either all 4 adducts (Na, K, H, 2Na) or solely the dominant sodium adduct peak are evaluated.

Table 1 additionally includes the results for copolyesters with 1,10-decanediol (DD), neopentyl glycol, and isophthalic acid (PES2–5). Although not the case for these specific copolyesters, increasing the number of monomeric repeating units might lead to wrong assignments of K, 2Na, and H adducts. This is caused by the exponentially increasing number of theoretical *m*/*z* values which at some point provides alternative polymer compositions for the actual K, 2Na, or H peaks. To avoid such mismatches, each polymer system has to be tested in this aspect prior to applying the simplification of only evaluating the dominant adduct peaks. Next to the abundancy of different terminating groups, it is of great interest to determine the ratio of incorporated comonomers to investigate the polycondensation reaction itself and further detect the structure–property relationships of a given polymer. *MALINTO* calculates the relative peak areas of the individual comonomers (%IPA, %NPG, %DD) which were further used to calculate the decanediol incorporation *x*_DD_ with eq 12. This *x*_DD_ value could then be directly compared to ^1^H NMR results. Details are given in the Supporting Information (Figure S1–S2, eq S1).
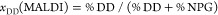
12

**Figure 5:**
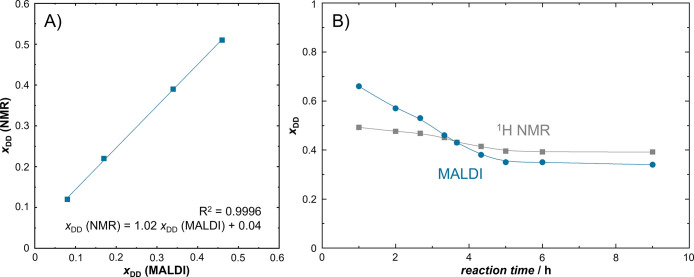
(A) Comparison of ^1^H NMR and MALDI results for 1,10-decanediol content *x*_DD_ in copolyesters with NPG and IPA. While absolute values are underestimated by the MALDI method, ^1^H NMR could be used for calibration (*R*^2^ = 0.9996). (B) During the course of a polycondensation reaction (PES4), *x*_DD_ values are useful for investigating monomer reactivities. Starting from dimers all esterified species are included in ^1^H NMR interpretation, whereas the composition of longer species is evaluated by MALDI (*m*/*z* = 600–4000).

**Figure 6:**
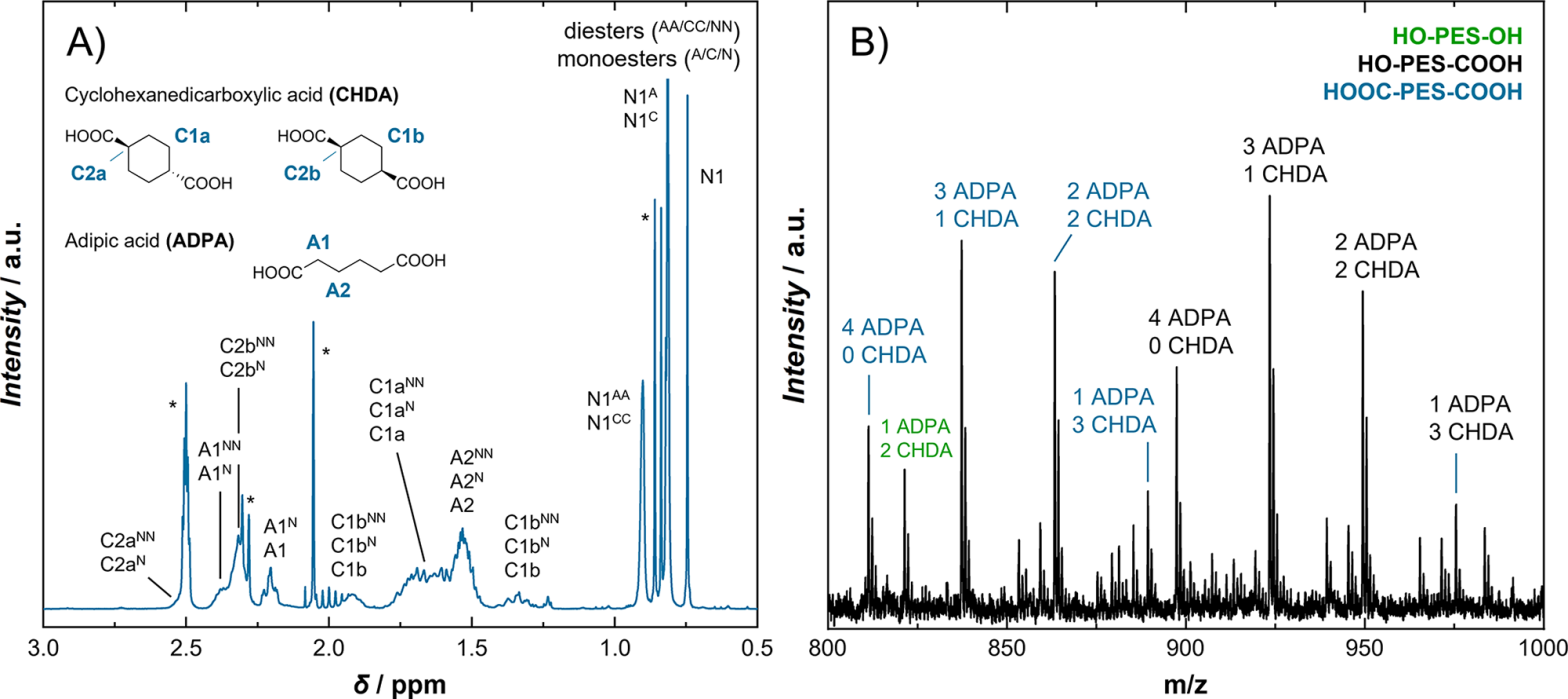
(A) Detail of ^1^H NMR for NPG-ADPA/CHDA copolyester PES6 after 2 h reaction time (intermediate product 04). Peak overlapping prevents determination of degree of esterification of acid components and, thus, determination of comonomer ratios. (B) In contrast, MALDI spectra give distinct peaks for different polyester compositions and allow determination of comonomer ratios.

As seen in Figure 5A and Table 2, MALDI and ^1^H NMR results for DD incorporation perfectly correlate for the investigated polyesters PES2–5, although absolute values are underestimated by the MALDI method. Such a phenomenon has been previously discussed for IPA-NPG copolyesters with maleic anhydride.^17^ While the investigation of comonomer ratios in polycondensates via ^1^H NMR seems advantageous in this case, peak overlapping prevents distinction of different species in some copolyesters (e.g., polyesters with 1,4-cyclohexanedicarboxylic acid described below, or branched polyesters in example 2). Additionally, peak overlapping in ^1^H NMR becomes more frequent while examining intermediate products because monomers, monoesters, and diesters often exhibit varying chemical shifts. An exemplary ^1^H NMR spectrum is given in Figure 6A.

**Table 2:** ^1^H NMR and MALDI Results for Decanediol Incorporation (*x*_DD_) of Different Copolyesters (PES2–5) Correlate Well as Shown in Figure 5

sample	*x*_DD_ (th)	*x*_DD_ (NMR)	*x*_DD_ (MALDI)
PES2	10	12	8 ± 0
PES3	20	22	17 ± 1
PES4	40	39	34 ± 4
PES5	50	51	46 ± 2

The polycondensation reaction of 1,10-decanediol, neopentyl glycol, and isophthalic acid was examined in more detail by collecting samples at different reaction times. Again, ^1^H NMR and MALDI methods were applied to investigate the comonomer ratios. As shown in Figure 5B, both methods agree that decanediol reacts faster than neopentyl glycol since *x*_DD_ is higher at the beginning and only stabilizes around the theoretical value of 0.4 after an approximate reaction time of 5–6 h. However, this effect is significantly more pronounced in the MALDI results. This can be explained by the different molecular weights observed by the two methods. While ^1^H NMR already includes dimers, only oligoesters are observed in the MALDI spectrum for which the *m*/*z* range has been set to 600–4000. The high *x*_DD_(MALDI) value at the beginning of the reaction supports the higher reactivity of decanediol since this comonomer is mainly found in the higher molecular weight fractions. A mistake due to the higher molecular mass of the DD monomer itself can be excluded since *x*_DD_ is evaluated for specific number of monomeric repeating units which is independent of the individual masses (Figure 3).

While IPA-NPG/DD copolyesters including intermediates can be investigated by both ^1^H NMR and MALDI, a cyclohexanedicarboxylic acid polyester (PES6) shall be given as an example in which the NMR method fails. As seen in Figure 6A, peak overlapping prevents quantification via NMR due to *cis*/*trans* isomerism of the monomer and again different shifts due to esterification. Peaks in the MALDI spectrum (Figure 6B) on the other hand are unambiguously assigned to polyester compositions revealing the number of individual monomeric repeating units. Thus, MALDI represents the ideal method for investigating the comonomer ratios of cyclohexanedicarboxylic acid (CHDA) and adipic acid (ADPA), especially in intermediate products for the evaluation of monomer reactivities. The course of a polycondensation reaction as shown in the Supporting Information (Figure S3) reveals the higher reactivity of the aliphatic adipic acid compared to CHDA. Such information can be used for the investigation of further (multifunctional) monomers and design of new synthesis procedures.

### Example 2: Branched Copolyesters and End-Capping

Using tri- or multifunctional monomers in a polycondensation reaction leads to branched polyesters with an increased number of free functionalities. These functional groups often provide certain properties of the polymer such as dispersibility in water or the ability for further cross-linking. Additional functionalities might be introduced by treating previously synthesized polycondensates with further reagents in an end-capping reaction. In the case of polyesters, trimellitic anhydride is widely used for end-capping to increase carboxylic acid moieties as terminating groups. Analysis of branched and/or end-capped polymers by ^1^H NMR causes difficulties due to the known reason for overlapping peaks (monomer, monoester, diester, triester, ...) as well as potentially low concentrations of the end-capping reagent.

**Figure 7:**
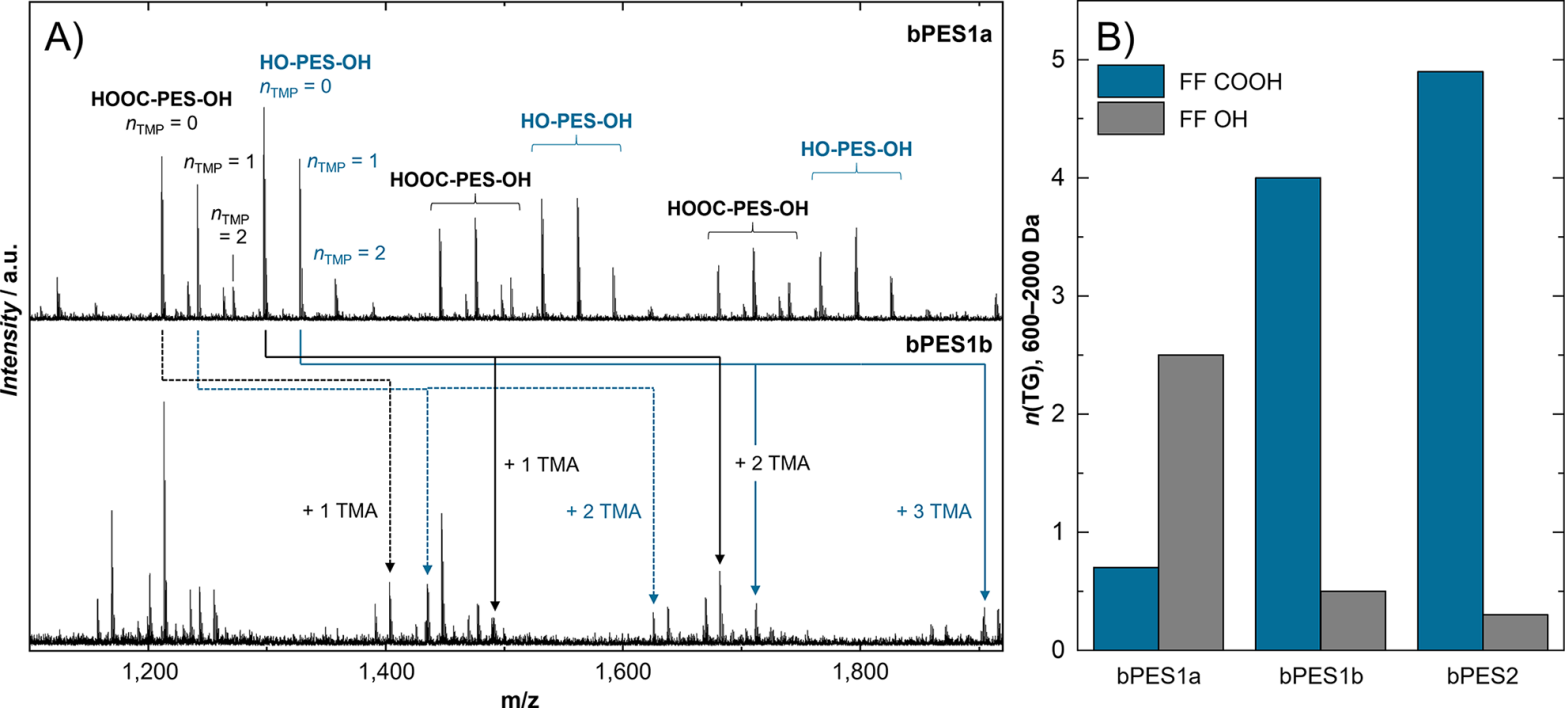
(A) MALDI mass spectra of branched polyesters (bPES1a,b) with trimethylolpropane (TMP) before and after end-capping with trimellitic anhydride (TMA). (B) The number and kind of free functionalities in a certain mass range can be used for comparing similar polyester systems produced under different reaction conditions.

In contrast, MALDI is a powerful tool for investigating such systems, as each branching monomer or successful end-capping changes the molar mass of the individual polymer chains (Figure 7A). Similar to previously discussed A_2_ + B_2_ polyesters, comonomer ratios can be determined as shown in Table 3. It has to be noted, however, that especially for branched polyesters the investigated *m*/*z* range is not necessarily representative for the whole sample. This is due to partial cross-linking leading to higher molecular weight species which lie outside applicable mass ranges in MALDI but can be investigated by size exclusion chromatography (Supporting Information, Figure S4). Comonomer compositions given in Table 3 show that MALDI results for the acid components resemble theoretical values given in brackets while the trimethylolpropane content seems to be underestimated. Since trimethylolpropane was reacted at temperatures up to 240 °C, cross-linking would be promoted and molecular masses are expected to be shifted outside the investigated *m*/*z* region. Trimellitic anhydride on the other hand was only left to react at 180 °C for 0.5 h, thus being less prone to cross-linking.

**Table 3:** Composition of Branched Polyesters Determined via MALDI Mass Spectrometry^a^

monomers	bPES1a	bPES1b	bPES2
Neopentyl glycol (%)	40 ± 1 (37)	36 ± 3 (30)	32 ± 4 (27)
Trimethylolpropane (%)	11 ± 2 (15)	8 ± 3 (12)	9 ± 3 (13)
Isophthalic acid (%)	49 ± 1 (48)	37 ± 2 (40)	33 ± 2 (33)
Trimellitic anhydride (%)		19 ± 2 (18)	25 ± 2 (27)
Free Functionalities			
Ø FF, 600–2000 Da	3.2	4.4	5.2
%FF COOH	22 ± 3	89 ± 3	95 ± 3

a Theoretical values are given in brackets. The number and ratio of free functionalities (FF) were calculated within a certain *m*/*z* range to provide good comparison of samples.

Since branching monomers are often introduced to provide more free functionalities in the polymer bulk, the Free Functionalities Statistics of *MALINTO* is of great interest. An average number of terminating groups (Ø FF) per polymer species in a certain *m*/*z* range provides a useful comparison of similar polyesters produced under different reaction conditions or in different batches (Figure 7B and Table 3). Although previously described limitations due to cross-linking equally apply and have to be kept in mind, MALDI presents the most applicable and informative method to evaluate the composition of branched polycondensates.

### Example 3: Multicyclic Polyesters

**Scheme 2:**
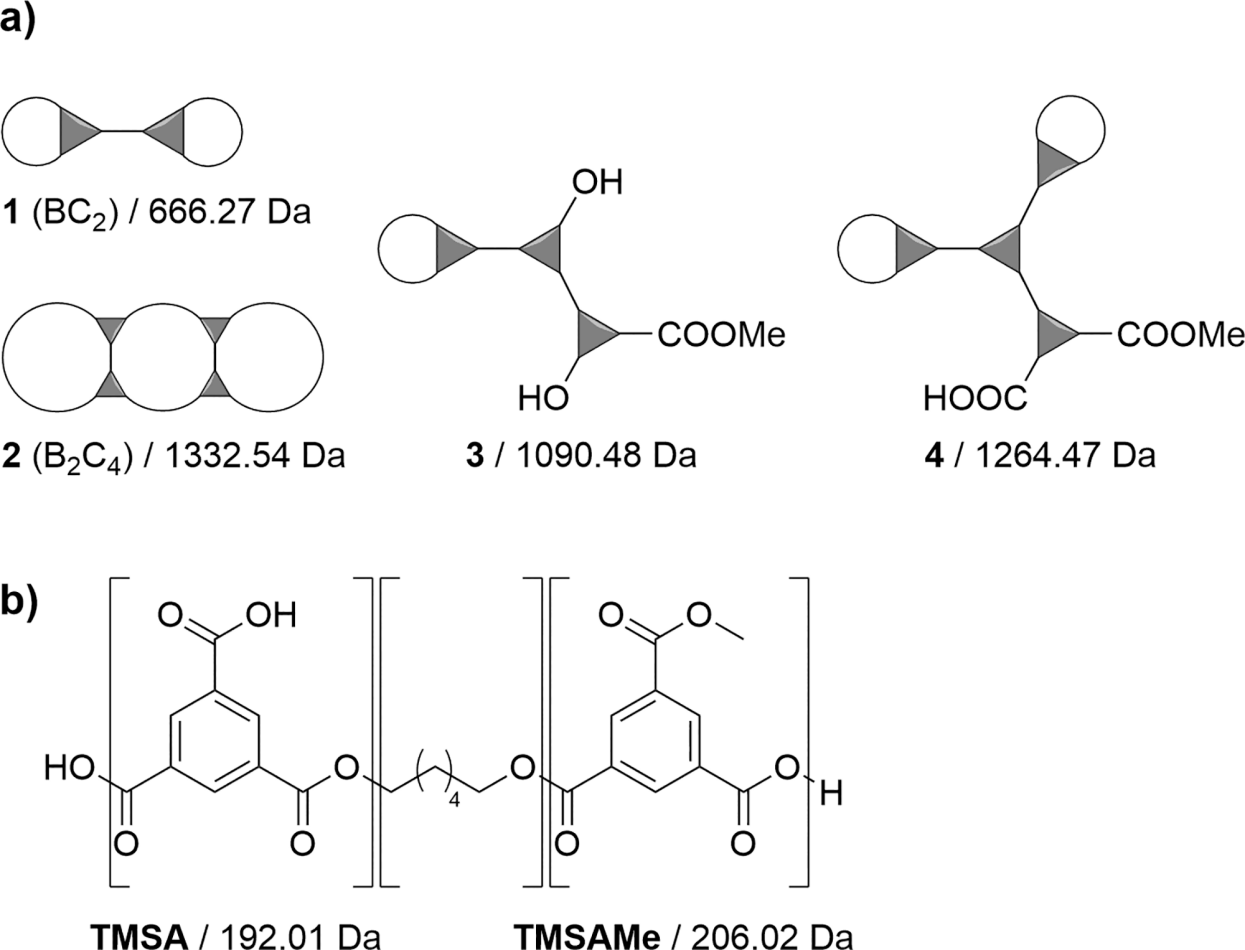
(a) Examples for (Multi)cyclic Oligoesters in Which Trimesic Acid (TMSA) Is Depicted as Triangle and Hexanediol as Line; (b) Masses of Such Structures Are Calculated Using TMSA Methyl Ester (TMSAMe) as a Third Monomeric Repeating Unit In the case of imperfect cycles, both acid and methylester terminating groups are present due to methanolic and hydrolytic work-up.

Inspired by the publication of Kricheldorf and Weidner about multicyclic polyesters,^33^ the functions of *MALINTO* were extended to calculate and assign masses of multicycles via special end group input data. Although trimesoyl chloride has been used for the synthesis with alkanediols, oligomeric masses are calculated like before using trimesic acid minus a water molecule (192.01 Da). As already discussed for A_2_ + B_2_ polycondensates, the cyclic (0 Da) and linear (18.01 Da) species are distinguished by the end group input data. Further cyclization in branched polyesters is caused by additional condensation reactions and, hence, the removal of water molecules (18.01 Da). The masses of multicycles can thus be calculated by defining additional end groups with nominal masses of −18.01 Da (2 rings), −36.02 Da (3 rings), etc. Examples for cyclic structures including masses from trimesic acid (TMSA) and hexanediol (HD) are given in Scheme 2a; the *m*/*z* of structure **2** (B_2_C_4_) is calculated according to eq 1 as
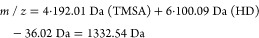


Depending on the synthesis, imperfect ring structures have also been detected via MALDI MS (structures **3** and **4** in Scheme 2a). Due to the methanolic and hydrolytic workup, both methyl esters or acid terminating groups are found instead of acyl chlorides. Additionally, hydroxyl terminating groups are possible. To achieve correct calculation of the polymer’s molecular weights, trimesic acid monomethyl ester (206.02 Da) was introduced as a third monomer (Scheme 2b). The *m*/*z* calculation of structure **4** is given as an example.
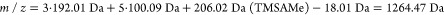
Finding the right input data for such complex perfect or imperfect (multi)cyclic structures might not be as straightforward as for the previously discussed examples. It is recommended to check resulting masses with a reference and/or by drawing chemical structures using suitable software which also calculates exact molecular masses. After identifying the correct input data, *MALINTO* efficiently decreases time for peak assignment and thus identification of polymer composition which is especially recommended for an increased number of analytes.

### Example 4: AB Polyesters and Chain-Growth Polymers

Finally, poly(lactic acid) and polystyrene with varying end groups shall be given as additional examples in the *MALINTO* application portfolio. Results for selected samples can be found in the Supporting Information (p. 6–7). It has to be noted that MALDI spectra of such simple polymers could also be semiautomatically interpreted using commercially available software. However, including these applications allows the exclusive use of *MALINTO* for the enhanced (semiquantitative) interpretation of MALDI spectra.

## Conclusion

While MALDI-ToF MS is a powerful technique for structure elucidation of polymers, several limitations like constrained resolution for broader *m*/*z* regions, varying ionization efficiencies, and dependence on sample preparation often prevent its use for quantification. Although previous studies showed potential for (semi)quantitative applications, the number of experiments is limited by the time-consuming interpretation of MALDI MS data. While several free and commercially available software packages give support for terminating group or copolymer composition of simple polymers, they cannot be applied to more complex systems such as A_2_ + B_2_ polycondensates synthesized from two different kinds of monomers. The newly developed *MALINTO* software facilitates and significantly accelerates the characterization of complex polymer structures including branched and multicyclic polycondensates while still fulfilling the needs during analysis of simple polymers.

*MALINTO* calculates a theoretical mass list from input data including the masses of the monomeric repeating units, end groups, and adducts. Three filters are applied to exclusively obtain realistic polymer compositions whose *m*/*z* values can further be compared to experimental MALDI data. The number of theoretical *m*/*z* values significantly increases with the complexity of the polymer structure which might lead to ambiguous assignments. Several tools are introduced to help clear the peak assignments, after which results are filtered according to comonomer and terminating group compositions in the Statistics tool. Results can be exported to Excel at each step, allowing individual interpretations and calculations of newly investigated polymer systems. *MALINTO* was successfully applied on several different examples, which shows the wide field of possible applications and aims to inspire fellow researchers to use MALDI mass spectrometry more extensively in general as well as specifically for quantitative applications.

## Supporting Information Available

Experimental procedures on polymer syntheses and characterization; Additional analytical data (MALDI, ^1^H NMR, SEC) on described polymer systems are provided as well as MALDI results for AB polyesters and chain-growth polymers (example 4) (PDF)

## Notes

The present manuscript describes the development, structure, and application of the free *MALINTO* software, which can be downloaded including a detailed step-by-step manual and examples at the following webpage: https://www.jku.at/en/institute-for-chemical-technology-of-organic-materials/publications/software/malinto/ (accessed on December 15, 2022).
