# Supplementary material for: MALINTO: A New MALDI Interpretation Tool for Enhanced Peak Assignment and Semiquantitative Studies of Complex Synthetic Polymers
Source: J Am Soc Mass Spectrom. 2023 Jan 4;34(2):293–303. doi: 10.1021/jasms.2c00311 (PMC9896554; doi:10.1021/jasms.2c00311)
Supplement: Supplementary file 1 — js2c00311_si_001.pdf [file js2c00311_si_001.pdf]

MALINTO: A new MALDI interpretation tool for enhanced peak assignment and semi-quantitative studies of complex synthetic polymers

Supporting Information

Klara M. Saller[1*], Daniel C. Pernusch[1], Clemens Schwarzinger[1]

[1]Institute for Chemical Technology of Organic Materials, Johannes Kepler University Linz, 4040 Linz, Austria / *corresponding author: klara.saller@jku.at

SI: EXPERIMENTAL

Monomers. For the synthesis of A_2+B_2 polyesters, neopentyl glycol (NPG) from Perstorp Holding AB, Sweden, and isophthalic acid (> 99 %) from Indorama Ventures Química S.L.U. were used. Further, 1,10-decanediol (99%) and adipic acid (99%) were purchased from Acros Organics, the isomeric mixture of 1,4-cyclohexanedicarboxylic acid from Eastman Chemical Company. The trifunctional monomers trimethylolpropane and trimellitic anhydride were provided by TIGER Coatings GmbH & Co. KG. These monomers were used for polyester synthesis without purification. Poly(lactic acid) was synthesized using L-lactide (98%) from Sigma Aldrich which was further purified via recrystallization in 2-propanol. Styrene (>99%, stabilized with 50 ppm hydroquinone) from Sigma Aldrich was extracted with an aqueous sodium hydroxide solution (10 *wt*%), washed with deionized water, dried with calcium chloride and distilled under reduced pressure.

Polyester synthesis. Polyester synthesis was performed in bulk using a 0.5 L reaction vessel and 4-neck lid equipped with a mechanical stirrer and a heating mantle. After melting the diol component(s), 0.9 mmol of a stabilizer, 1.1 mmol of a tin catalyst, the acid components, and a retainer were added. The reaction mixture was heated under inert atmosphere using different temperature programs as summarized in Table S1. Water formed during the polycondensation was collected in a Dean-Stark trap. After 5 h, the pressure was reduced to 500 mbar. After another hour, the entrainer was removed and the mixture left to react further at 50 mbar for 3 h. Finally, the hot polyester melt was poured into aluminum dishes to cool down.

For branched polyesters, 2- and 3-step syntheses were performed to avoid extensive crosslinking and gelation of the resins. For bPES2, neopentyl glycol, isophthalic acid and trimethylolpropane were stepwise heated up to 240 °C until the clearing point of the reaction mixture. This indicated a nearly full conversion of isophthalic acid. After cooling down to 160 °C, trimellitic anhydride was added for the endcapping reaction. For bPES1, a precondensate of isophthalic acid (85% of total mass) and neopentyl glycol was synthesized using again the clearance point as an indication of full conversion. In a second step, trimethylolpropane and the remaining 15% of isophthalic acid were reacted and then, trimellitic anhydride was added in a third step. All steps were carried out under inert atmosphere under ambient pressure.

For poly(lactic acid) (**PLA**) synthesis 1 g of recrystallized lactide (6.9 mmol) was transferred to a two-neck round bottom flask connected to a Schlenk line which has previously been evacuated and purged with Argon three times. 0.05 mmol of tin(II)-2-ethylhexanoate (Alfa Aesar, technical purity) was used as catalyst and added as a 0.2 mol L^{-1} solution in absolute toluene. Methanol (VWR, 100%) was tested for initiation of the ring-opening polymerization and added to the reaction flask after dilution with toluene (1 *wt*% methanol/lactide). The medium was stirred, heated to 180 °C and kept at this temperature for 1 h while the sample solidified. After slightly cooling down, 5 mL of chloroform was added and the polymer dissolved under reflux. The polymer was further precipitated by dropwise addition of 15 mL of methanol. The precipitate was filtered, washed and dried in a vacuum drying cabinet at 40 °C.

Table S1: Composition of A_2+B_2 polycondensates PES1–6 and branched polyesters bPES1–2 including individual temperature programs. NPG…neopentyl glycol, IPA…isophthalic acid, DD…1,10-decanediol, ADPA…adipic acid, CHDA…1,4-cyclohexandicarboxylic acid, TMP…tri-methylolpropane, TMA…trimellitic anhydride.

Polymer	Monomers (*mole fractions* / %)	Temperature Program
**PES 1**	NPG (0.49), IPA (0.51)	2 h 180 °C; 1 h 200 °C; 1 h 220 °C; 5 h 240 °C
**PES 2**	NPG (0.44), DD (0.05) / IPA (0.51)	
**PES 3**	NPG (0.39), DD (0.10) / IPA (0.51)	
**PES 4**	NPG (0.29), DD (0.19) / IPA (0.51)	
**PES 5**	NPG (0.24), DD (0.24) / IPA (0.52)	
**PES 6**	NPG (0.49) / ADPA (0.26), CHDA (0.26)	1 h 140 °C; 1 h 160 °C; 1 h 170 °C; 2 h 180 °C; 4 h 200 °C
**bPES1a**	NPG (0.37), TMP (0.15) / IPA (0.48)	1 h 160 °C; 0.7 h 200 °C; 0.7 h 220 °C; 1.3 h 240 °C (clearance);<br>0.3 h 160 °C; + TMP; 0.7 h 180 °C; 1 h 200 °C; 0.7 h 220 °C (clearance)
**bPES1b**	NPG (0.30), TMP (0.12) / IPA (0.40), TMA (0.18)	**bPES1a** + 0.3 h 160 °C; + TMA; 0.5 h 180 °C
**bPES2**	NPG (0.27), TMP (0.13) / IPA (0.33), TMA (0.27)	1 h 160 °C; 0.7 h 200 °C; 0.7 h 220 °C; 0.3 h 240 °C (clearance);<br>0.3 h 160 °C; + TMA; 0.5 h 180 °C

Polystyrene synthesis. Polystyrene was synthesized via anionic polymerization using *n*-butyl lithium as initiator (Sigma Aldrich, 1.6 M in hexanes). The procedure was carried out under inert conditions on a Schlenk line. 45 mL absolute tetrahydrofuran was cooled to -78 °C, 4 mL (34 mmol) of purified styrene and 2.2 mL (3.5 mmol) of the butyl lithium solution were added subsequently. After 1 h 10 *vol*% of the reaction mixture was withdrawn and precipitated in methanol. This intermediate product (**PS**) was filtered and dried at 60 °C overnight. In the meantime, 1 mL styrene was added to the reaction medium. After another hour, the reaction was terminated by adding 1.3 mL ethylene oxide solution (Sigma Aldrich, 2.5–3.3 M in tetrahydrofuran). The product (**PS-OH**) was again precipitated in methanol, isolated by centrifugation and dried.

MALDI-ToF MS. Samples were analyzed via MALDI mass spectrometry as described in the main text. Variations of solvents, matrices and salts are given in Table S2.

Table S2: MALDI sample preparation of different polymer species (DHB…2,5-dihydroxybenzoic acid, DCTB… *trans*-2-[3-(4- *tert.*-butylphenyl)-2-methyl-2-propenylidene]malononitrile).

Polymer	Solvent	Matrix	Salt
**PES1–6**	Tetrahydrofuran (THF)	DHB	CF_3COONa (CF_3COOK)
**bPES1,2**	THF	DHB	CF_3COONa
**cPES1,2**	$CHCl_3$	DHB, DCTB	CF_3COOK
**PLA**	$CHCl_3$	DCTB	CF_3COONa
**PS, PS-OH**	THF	Dithranol	$AgClO_4$

Nuclear magnetic resonance. ^{1}H NMR spectra were recorded on a Bruker Avance 300 MHz spectrometer. Polyester samples PES1–5 were dissolved in deuterated chloroform, for the aliphatic PES6 deuterated dimethyl sulfoxide was used as NMR solvent. Concentrations around 25 mg mL^{-1} were chosen. Peaks are given in ppm relative to tetramethylsilane.

Size exclusion chromatography. Size exclusion chromatography (SEC) of PES1–6 and branched polyesters were measured in tetrahydrofuran on a setup including an HPLC pump (PU-2086 Plus, Jasco), an autosampler (728, Bischoff), and three detectors (UV: UV-975, Jasco, wavelength: 230 nm for aliphatic and 260 nm for aromatic samples; refractive index: 200, Perkin Elmer; light scattering: MiniDAWN, Wyatt Technology). Phenogel columns with pore sizes of 50, 500, and 10^4 Å were used for separation at 40 °C. Samples were dissolved in THF at concentrations of 2–3 mg mL^{-1}. Calibration was performed with polystyrene standards. Molecular masses of samples (number average, M_n, mass average, M_w) as well as the polydispersity index were calculated as polystyrene equivalents.

SI: RESULTS

Example 1: A_2+B_2 homo- and copolyesters. Figure S1 shows the detail of an ^{1}H NMR spectrum of a copolyester synthesized with 1,10-decanediol (DD), neopentyl glycol (NPG) and isophthalic acid (IPA). The degree of esterification for long aliphatic monomers such as decanediol was estimated by two separated signals for D2 protons. Before esterification these protons found in the region of 1.65–1.50 ppm, after esterification a peak around 1.85–1.65 ppm appeared. The latter was compared to mono- and diester peaks of neopentyl glycol (diester N2^{II}: 1.20–1.05 ppm, monoester N^{I}: 1.05–0.95 ppm). Since the degree of esterification of a monoester is only 50%, half of the respective integral is used for the calculation of x_{DD} as shown in equation S1. x_{DD} was used for comparing MALDI with ^{1}H NMR results and investigation of 1,10-decandiol and neopentyl glycol reactivities.

$$x_{DD}(\mathrm{NMR}) = Area\ (\mathrm{DD}, 1.85-1.65\ \mathrm{ppm})\ /\ [Area\ (\mathrm{DD}, 1.85-1.65\ \mathrm{ppm}) + Area\ (\mathrm{NPG^{II}}, 1.20-1.05\ \mathrm{ppm}) + Area\ (\mathrm{NPG^{I}}, 1.05-0.95\ \mathrm{ppm})/2] \qquad (S1)$$

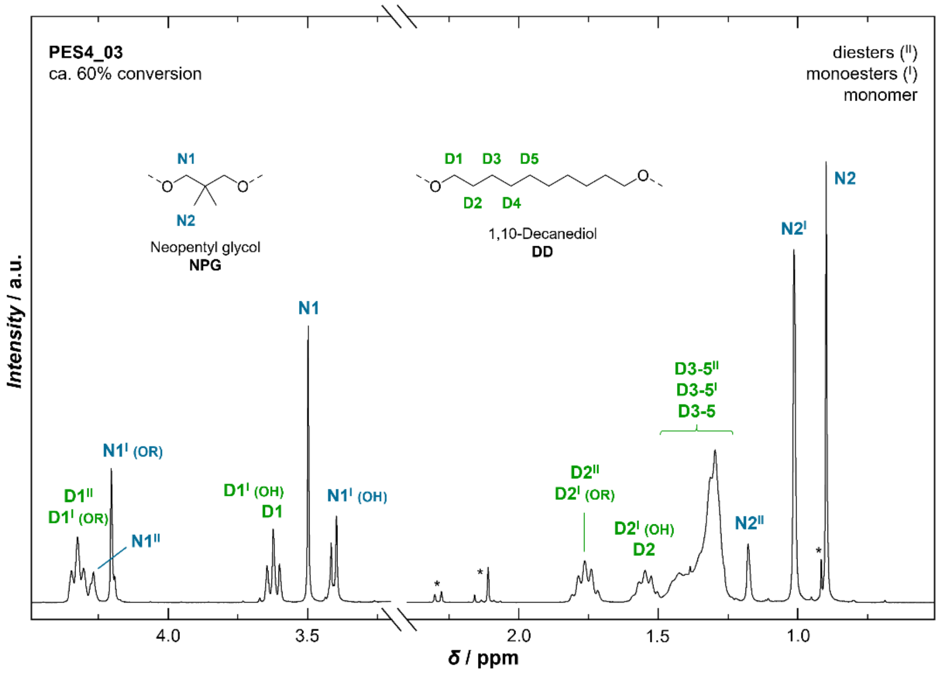

Figure S1: Detail of ^{1}H NMR for NPG/DD-IPA copolyester PES4 after 2 h reaction time (intermediate product 03). Isolated peaks for both decanediol (1.83–1.66 ppm) and neopentyl glycol (1.21–0.95 ppm) were used for quantification. DD polyester peak includes half of the monoester species.

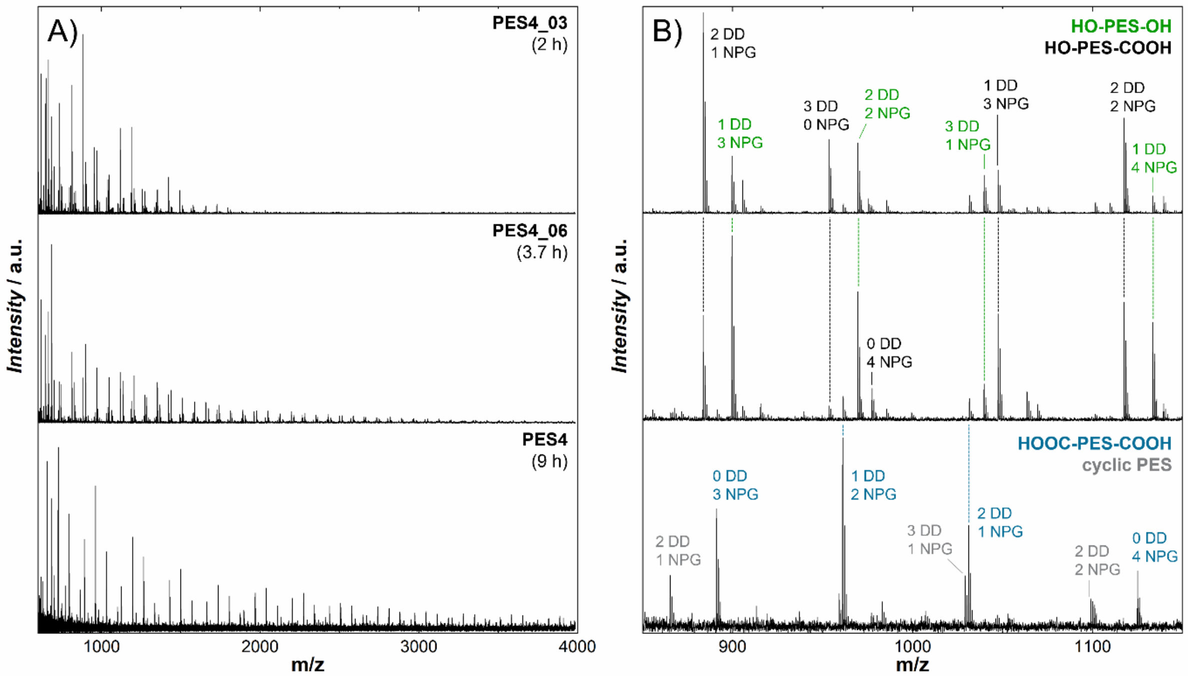

Figure S2: A) Overview and B) detail of MALDI mass spectra of NPG/DD-IPA copolyester PES4 and intermediates. Different series of terminating groups are highlighted in the colors green (double OH terminated), black (mixed terminated), turquoise (double COOH terminated) and grey (cycles).

The MALDI mass spectra of NPG/DD-IPA copolyester PES4 including the intermediate products after 2 h (03) and 3.7 h (06) reaction time are shown in Figure S2. While molecular mass distributions were shifted to higher m/z values during the course of the reaction, also different kind of polyester species were found in the MALDI spectra. At the beginning, decanediol seems to be the predominant diol in the sample composition although DD probably reduced the ionization efficiency of the analyte (Figure 5, main

text) leading to an underestimation of x_{DD}. Additionally, different ratios of terminating groups were observed. Towards the end of the reaction, double carboxyl terminated chains dominated the spectra due to the excess of acid used in the synthesis while both mixed and double hydroxyl terminated species were found at the beginning of the polycondensation process. Ring formation only occurred at advanced reaction times of 6 h or longer.

While degree of esterification and thus comonomer ratios could be determined for many polyesters using ^{1}H NMR, peak overlapping prevented necessary deconvolution for several other systems. One example were copolyesters containing 1,4-cyclohexandicarboxylic acid (CHDA) as described in the main text. In contrast to ^{1}H NMR, MALDI measurements revealed the polyester composition and thus incorporation of CHDA compared to adipic acid (ADPA). By performing such investigations during the course of a polycondensation reaction, a higher reactivity of adipic acid was observed while CHDA only slowly incorporated into the polyester structure (Figure S3). Due to different ionization efficiencies comonomer ratios obtained from MALDI MS might not represent absolute values which is, however, not relevant in the shown example which shows a trend during the polyesterification of the same monomer mixture.

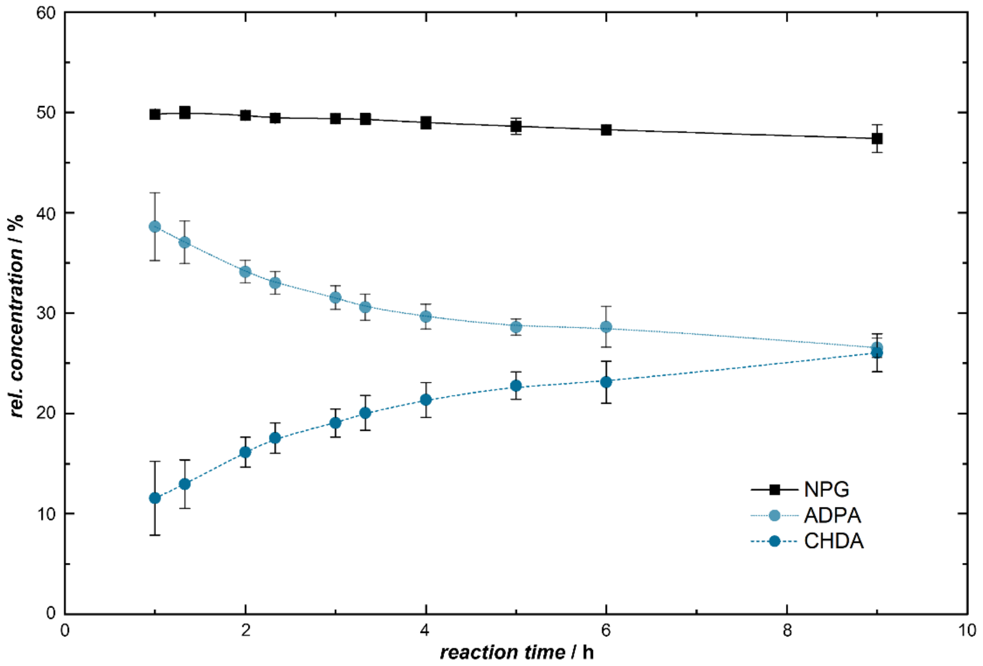

Figure S3: Comonomer ratios of PES6 intermediate products determined via MALDI MS show a higher reactivity of adipic acid (ADPA) compared to 1,4-cyclohexanedicarboxylic acid (CHDA).

Example 2: Branched copolyesters and endcapping. Besides the crucial advantage of distinct peaks for branched and endcapped polyesters in MALDI mass spectrometry, a good resolution can only be obtained in a limited spectral width which does not necessarily represent the overall polyester composition. Thus, comparison of branched polyesters is only recommended for similar systems while samples should additionally be investigated via size exclusion chromato-graphy. Size exclusion chromatograms of discussed bPES are shown in Figure S4. While peak patterns vary in the low molecular weight region which is mostly included in the MALDI mass spectra (600–4000 Da), the

main peak shapes are similar as are calculated molecular weights. Additional to the findings presented in the main text, this confirms the suitability of the MALDI method.

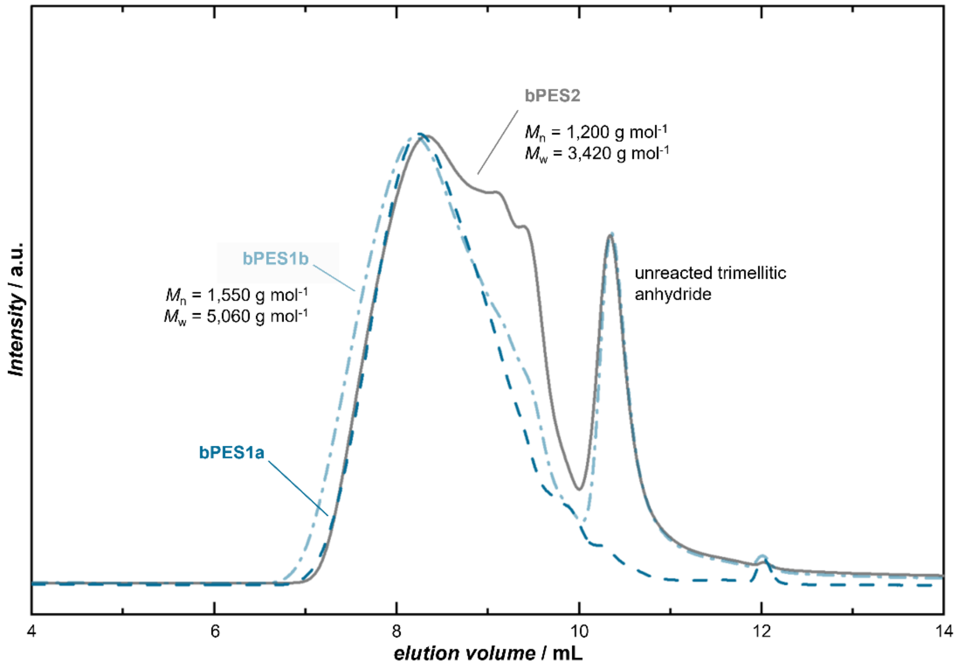

Figure S4: Size exclusion chromatograms of branched polyesters bPES1 (a - before endcapping, b - after endcapping) and bPES2. The peak around 10.3 mL appears after endcapping and is caused by unreacted trimellitic anhydride/acid.

Example 4: AB polyesters and chain-growth polymers. A prominent example of an AB polyester based on a hydroxycarboxylic acid is poly(lactic acid). As for other polycondensates, the mass of the repeating unit equals the monomer mass minus water. However, no second monomer with a different kind of functionality is required because the monomer already carries a carboxylic acid and a hydroxyl group. Therefore, this class of polycondensates can be treated similar to chain-growth polymers like polystyrene. Since polystyrene forms via a polymerization reaction, the repeating unit equals the monomer mass. The functionality fields are both filled with the number of double bonds present in the monomer (e.g. 1 for styrene, 2 for divinylbenzene). A screenshot of the input data is given in Figure S5.

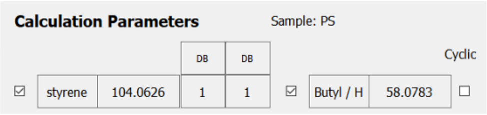

Figure S5: Input data for chain-growth polymers such as polystyrene.

The MALDI mass spectra and end group statistics of a poly(lactic acid) and a polystyrene sample are given in Figure S6 and S7, respectively. Poly(lactic acid) was synthesized via ring-opening polymerization of lactide. Although methanol has been used as initiator, MALDI MS revealed a second series of PLA initiated with propanol. Residuals of 2-propanol are expected to cause these byproducts because this solvent has been used for the recrystallization of the monomer. Although the ratio of the initiators does not necessarily present absolute values as has been demonstrated for copolyesters, it is

constant for chain lengths between 25 and 50 repeating units which confirms simultaneous initiation. The lack of propanol-initiated PLA at lower chain lengths can be explained by the decreasing signal intensity which affects the lower concentrated species more severely until the peak area cannot be determined anymore.

A different behavior was observed for a polystyrene sample synthesized via anionic polymerization and succeeding termination with ethylene oxide. The ratio of successfully OH terminated chains increased with the chain length. This could be attributed to the very sensitive anionic polymerization which is terminated by any kind of impurities, for example moisture. If chains are terminated accidentally via protonation, polystyrene can neither react with further monomers to increase the molecular weight nor react with the later introduced terminating agent. Thus, the trend suggests efficient termination while non-functionalized polystyrene is present due to premature termination.

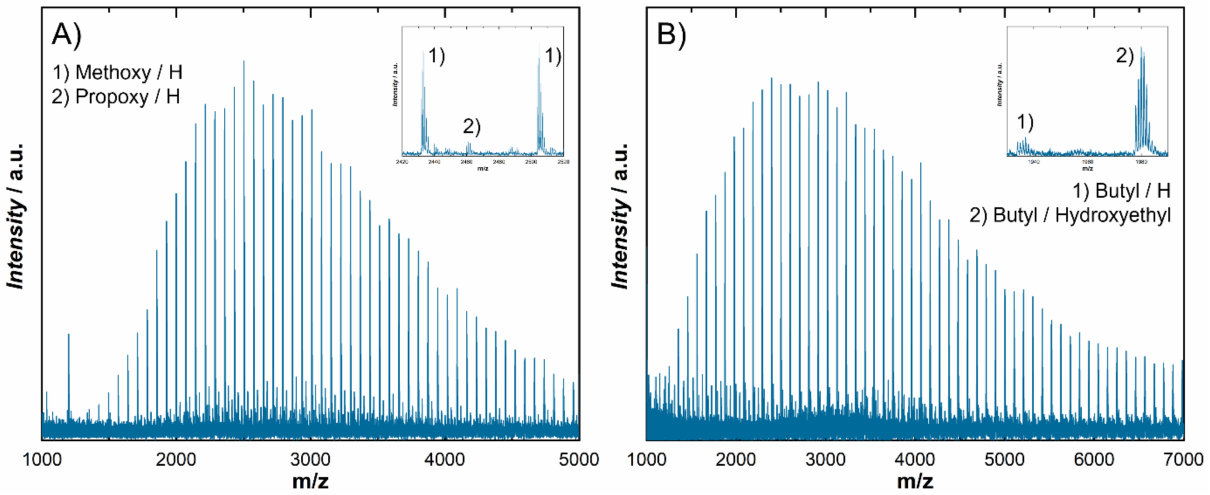

Figure S6: MALDI mass spectra of A) poly(lactic acid) with methanol and propanol initiation, and B) polystyrene with proton and ethylene oxide termination. Structures and statistics are given in Figure S7.

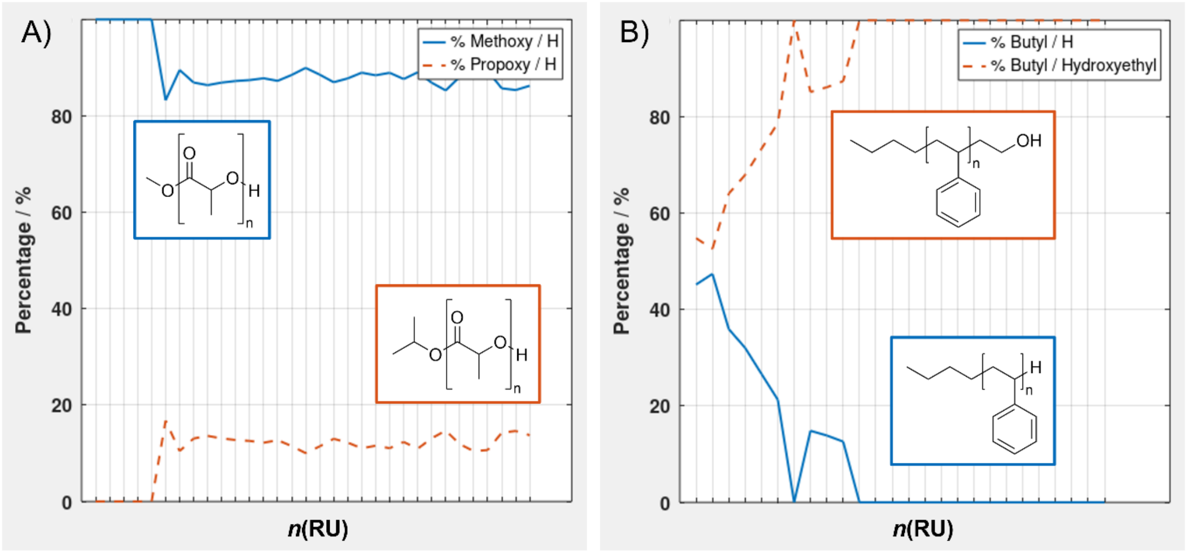

Figure S7: End group statistics for A) poly(lactic acid) synthesized via ring-opening polymerization of lactide using methanol as initiator and for B) polystyrene synthesized via anionic polymerization using *n*-butyl lithium as initiator and ethylene oxide as terminating agent.
